# Morbidity Rates in an Area with High Livestock Density: A Registry-Based Study Including Different Groups of Patients with Respiratory Health Problems

**DOI:** 10.3390/ijerph17051591

**Published:** 2020-03-01

**Authors:** Christos Baliatsas, Michel Dückers, Lidwien A.M. Smit, Dick Heederik, Joris Yzermans

**Affiliations:** 1 Department of Disasters and Environmental Hazards, Netherlands Institute for Health Services Research (NIVEL), 3513 CR Utrecht, The Netherlands; M.Duckers@nivel.nl (M.D.);; 2 Institute for Risk Assessment Sciences (IRAS), Utrecht University, 3584 CM Utrecht, The Netherlands; L.A.Smit@uu.nl (L.A.M.S.);

**Keywords:** livestock, public health, epidemiology, respiratory health, morbidity, general practice

## Abstract

There is continuing debate and public health concern regarding the previously confirmed association between high livestock density and human health. The primary aim of the current study is to assess the prevalence of respiratory and other health problems in a livestock dense area in the Netherlands, based on recent longitudinal health data and a large sample. Analyses are expanded with the investigation of different subgroups of patients with respiratory health problems and the inclusion of various chronic and acute health outcomes, as well as prescribed medication. Prevalence of health symptoms and chronic conditions was assessed for the period 2014–2016, based on electronic health records registered in 26 general practices located in areas with intensive livestock farming in the Netherlands (“livestock dense area”, n = 117,459 unique residents in total). These were compared with corresponding health data from general practices (n = 22) in different rural regions with a low density of livestock farms or other major environmental exposures (“control area”, n = 85,796 unique residents in total). Multilevel regression models showed a significantly higher prevalence of pneumonia in the total sample in the livestock dense area, which was also observed among susceptible subgroups of children, the elderly, and patients with chronic obstructive pulmonary disease (COPD). Lower respiratory tract infections, respiratory symptoms, vertigo, and depression were also more common in the livestock dense area compared to the control area. In general, there were no significant differences in chronic conditions such as asthma, COPD, or lung cancer. Prescription rates for broad-spectrum antibiotics were more common among patients with pneumonia in the livestock dense area. Acute respiratory infections and symptoms, but not chronic conditions, were considerably more common in areas with a high livestock density. Identification of causal pathogens on the basis of serological analyses could further elucidate the underlying mechanisms behind the observed health effects.

## 1. Introduction

It is well-documented that agriculture constitutes a major source of air pollution [1,2,3,4]. Livestock farms emit high levels of environmental agents and pathogens that could elicit adverse health effects, such as bacteria, viruses, endotoxins, and particular matter [5]. There is an increasing body of evidence suggesting an association between livestock animals and the zoonotic disease transmissions [6]. Although characterization of livestock exposure has been challenging due to lack of information regarding exposure type and levels in the proximity of livestock farms [5], several epidemiological studies have investigated health effects in residents living in the neighborhood of livestock farms, such as respiratory complaints, pneumonia, asthma, allergies, and lung function deficit [7,8,9,10,11,12]. In the Netherlands in particular, the magnitude of the risk for adverse effects can be large, considering the high animal density alongside with the high human population density [4]. A representative example is the Q-fever outbreak caused by the bacterial pathogen *Coxiella burnetii*, affecting more than 4000 people in the period 2007–2010 [13], with dairy goats and dairy sheep being the main source of the pathogen.

Following that incident, the potential health risks of living in the vicinity of large livestock farms has been receiving increased attention, due to a number of findings regarding the increased rates of primarily respiratory symptoms and infections in the eastern part of the province of North Brabant and the northern part of the province of Limburg. More specifically, the prevalence of primary care-registered pneumonia and other lower respiratory tract infections, as well as chronic bronchitis, was found to be consistently higher in livestock dense areas compared to areas with low livestock density [14,15]; a higher prevalence of nonrespiratory conditions such as atopic eczema was also observed in both young children and adults [14]. Analyses in the same area including individual estimates of livestock exposure in relation to primary care-registered data have also demonstrated that living near goat and/or poultry farms is a risk factor for pneumonia [9,14,16] and also Q-fever [9,14]. Other findings also indicate a possible impact of livestock exposure on the health status of patients with compromised respiratory health; these showed increased respiratory symptoms and more exacerbations among chronic obstructive pulmonary disease (COPD) patients residing in livestock dense areas [17,18], while livestock exposure was also associated with respiratory symptoms in patients with overlapping diagnoses of asthma and COPD [19].

These previous findings reflect the need for regular health-monitoring in areas with high livestock density. The aforementioned studies were based on data up to the year 2013. Based on more recent longitudinal health data, the aim of the present study is to expand on previous studies with the investigation of different subgroups, such as children, elderly, and patients with respiratory health problems, as well as the inclusion of various chronic and acute health outcomes and prescribed medication.

## 2. Materials and Methods

### 2.1. Study Design and Participants

An observational study was conducted in the Netherlands, based on longitudinal health data from electronic health records (EHR) obtained for the period 2014–2016 from 48 different general practices in total (n = 26 located in the “livestock dense area” and n = 22 in the “control area”) in the Primary Care Database (PCD) of the Netherlands Institute for Health Services Research (NIVEL) [20]. Every resident is obliged to be registered at one practice, and general practitioners (GPs) act as gatekeepers to secondary care. The number of included practices and registered people per year are shown in Table A1 (Appendix A).

The “livestock dense area” refers to a highly populated livestock-dense rural region in the eastern part of the province of North Brabant and the northern part of the province of Limburg (see graphical abstract). For instance, based on estimates for the study population from provincial databases on compulsory environmental licenses, about one-third of the residents lived within 2000 m from a goat farm, while more than 90% lived within 1000 m from cattle, within 1500 m from pig, or within 2000 from poultry farms. High density of livestock farms in the selected areas has been well-documented in previous studies as well [15,21]. Furthermore, livestock farms are spatially associated with ambient endotoxin concentrations in these areas [22,23].

The “control area”, on the other hand, refers to other rural areas in the Netherlands with substantially lower livestock farm density, including the provinces of North Holland, South Holland, Utrecht, Drenthe, Gelderland, Friesland, Zeeland, and Groningen, as well as the western part of North Brabant and the southern part of Limburg. In the selected areas, there were generally no other known major landscape features that could affect residents’ health [15].

### 2.2. Ethics

The NIVEL Primary Care Database (PCD) complies with the regulations of the Dutch Data Protection Authority and the Dutch law regarding use of health data for epidemiological research purposes (Dutch Civil Law, Article 7:458). Medical information, as well as address records, were kept separated with the support of a Trusted Third Party (“Stichting Informatie Voorziening Zorg: IVZ”, Houten, The Netherlands).

### 2.3. Definition of Subgroups

The present study focused on the general population living in the investigated areas, as well as on several susceptible subgroups, namely, children, elderly persons, and respiratory patient subgroups (pneumonia, asthma, COPD, overlapping diagnoses of asthma and COPD/asthma and COPD overlap syndrome; ACOS). Each case definition is explained in Appendix A (Table A2).

### 2.4. Health Outcome Assessment

The investigated health outcomes were registered by the GPs following the International Classification of Primary Care (ICPC) [24]. Prevalence estimates were based on care episodes; each episode contains all patient encounters within an ICPC code [25]. The health outcome data represent prevalence of three classifications of health outcomes: chronic (irreversible) disorders, long-lasting (reversible) conditions, and acute conditions. A “symptom-free” period is taken into account that determines whether ICPC records belong to the same episode. For instance, respiratory infections such as pneumonia are classified as an acute condition, meaning that the episode has an “end” after a certain “symptom-free” period (e.g., 3 months), while episodes for chronic disorders remain “open”, since these concern irreversible conditions (e.g., COPD). In the current episode construct, health complaints were not “overruled” by chronic disorders (e.g., for the purpose of the present study, ”coughing” that fell within an episode of COPD was considered as a separate episode). Selection of health outcomes was based on potential relevance to livestock exposure, as assessed in previous investigations [15] (see Appendix A, Table A3). Moreover, prevalence of registered medication related to broad-spectrum antibiotics that are often prescribed for the treatment of respiratory tract infections (amoxicillin and doxycycline), as well as all prescriptions within the broader cluster of “anti-infectives for systemic use” (antibacterials, antimycotics, antimycobacterials, antivirals, immune sera/immunoglobulins, and vaccines), were also examined as outcome variables, classified according to the anatomical therapeutic chemical classification system (ATC) [26].

### 2.5. Statistical Analyses

Multilevel logistic regression analyses were carried out, taking into account the hierarchical structure of the data (registered people nested within general practices). The dependent variable in all analyses was the annual prevalence of the examined health outcomes, while the independent variable was the group type (study vs. control group). All analyses were adjusted for gender and age (polynomial, in order to allow for a potential nonlinear trend between age and morbidity) and registry duration. For each comparison, odds ratios (OR) with 99% confidence intervals (CI) were computed. A *p*-value of <0.01 was considered statistically significant, to control for multiple testing. In addition, sensitivity analyses were performed by repeating the main analyses after adjusting for a socioeconomic status (SES) score (based on the year 2016), provided by the Netherlands Institute for Social Research at a four-digit postal code level (PC4) [27]. This was based on the average household income, proportion of low family incomes, percentage of low-educated residents, and unemployment rates among residents [27,28]. Analyses were performed with STATA version 15.0 (StataCorp LP, College Station, TX, USA).

## 3. Results

### 3.1. Sample Characteristics

Sample characteristics in the study and control group, including different subgroups, are shown in Table A4 (Appendix A). Demographic characteristics, such as age and gender, did not significantly differ between the study and control area in the total sample. Average prevalence of the examined health conditions is presented in Table A5 (Appendix A). Upper respiratory tract infections; eczema; asthma; respiratory symptoms (cough, shortness of breath/dyspnea, and wheezing); and also, hypertension and coronary heart disease (among people ≥ 40 years old), were the most common health conditions on average in the study and control area. Furthermore, prevalence of prescriptions for anti-infectives in the livestock dense area was 20.4% in 2014, 20.2% in 2015, and 19.2% in 2016, while the use of broad-spectrum antibiotics was 8.4%, 8.61%, and 7.62%, respectively. In the control area, prevalence of anti-infective prescriptions was 21.2% (2014), 22.2% (2015), and 20.5% (2016), while for broad-spectrum antibiotics, this was 8.91%, 9.84%, and 8.1%, respectively.

### 3.2. Differences in Prevalence Rates between Areas with High and Low Livestock Density

Analyses in the total sample showed consistently significant differences in the prevalence of pneumonia for all years (Table 1). The same was observed for vertiginous syndrome. Moreover, the annual prevalence of lower respiratory tract infections (including pneumonia) was significantly higher in the livestock dense areas in 2015 and 2016.

Acute respiratory symptoms (cough, dyspnea, and wheezing) and depression were also more common in the dense areas but only statistically significant in one year (Table 1). Prevalence of chronic bronchitis/bronchiectasis was clearly higher in the exposed, although not statistically significant.

On the other hand, asthma tended to be less common in the livestock dense areas, but again, this difference was not significant. All observed findings remained robust after adjusting for SES, except for pneumonia in 2014, which became borderline nonsignificant (OR = 1.40, 99% CI 0.99–2.00, *p* = 0.014).

### 3.3. Differences in Health Outcomes within Respiratory Patient Subgroups

As shown in Table 2, the prevalence of asthma and upper respiratory tract infections was significantly lower in the livestock dense areas in 2014 and 2015, respectively, among patients with pneumonia. Rates of other symptoms and conditions did not differ between the two areas.

Among asthma patients (without comorbid COPD), no statistically significant findings were observed. However, in area differences in pneumonia, the broader cluster of lower respiratory tract infections (including pneumonia) and acute respiratory symptoms seemed to increase in the course of time (Table 3); the latter became significant when analyses were repeated without excluding patients with comorbid COPD from the group (Appendix A, Table A12).

Area differences among COPD patients (without comorbid asthma), on the other hand, appeared to be more pronounced, as indicated by the generally larger ORs (Table 4). There were, consistently, significantly higher rates for pneumonia, lower respiratory tract infections, vertiginous syndrome, and depression (years 2015 and 2016). When COPD patients with comorbid asthma were not excluded from the analyses, between-area differences were more pronounced regarding acute respiratory symptoms (Appendix A, Table A15), instead of pneumonia and lower respiratory tract infections.

No significant differences were found between the study and control area among patients with overlapping diagnoses of COPD and asthma (ACOS), except for an increasing difference in respiratory symptoms that reached statistical significance in 2016 (Appendix A, Table A16). On the other hand, when the ACOS group was defined as any patient with asthma or COPD, significantly higher rates were observed in the livestock dense area for pneumonia, lower respiratory tract infections, acute respiratory symptoms, vertiginous syndrome, and depression, especially in the years 2015 and 2016 (Appendix A, Table A17). The observed differences within the examined respiratory patient subgroups remained generally consistent after adjusting for SES (data not shown).

### 3.4. Differences in Health Outcomes within Susceptible Age Subgroups

Prevalence of pneumonia (primarily bronchopneumonia) was generally more common among children in the livestock dense areas and differed significantly compared to the control group in 2016 (Appendix A, Table A6). Results remained consistent after adjusting for SES (OR = 2.15, 99% CI 1.18–3.90). Additionally, although not statistically significant, between-area differences in chronic enteritis and vertiginous syndrome seemed to become larger over the examined years.

There were more consistent significant differences between areas in the elderly group (≥65 years) (Appendix A, Table A7). The annual prevalence of pneumonia, lower respiratory tract infections, and respiratory symptoms was significantly higher in the livestock dense areas in 2015 and 2016. In addition, a significantly higher prevalence of vertiginous syndrome was observed for all years and higher depression rates for 2014 and 2015 (Appendix A, Table A7). Inclusion of SES in the regression model did not alter the results.

Analyses on age subgroups of respiratory patients did not yield any consistent differences between the study and control area (Appendix A, Table A8, Table A9, Table A10 and Table A11).

### 3.5. Differences in Prescription of Broad-Spectrum Antibiotics and the Greater Cluster of Anti-Infectives for Systemic Use

A detailed overview of the results is given in Table 5. Although prescription rates in the total sample and most of the investigated subgroups were higher in the control area, those for broad-spectrum antibiotics were more common among patients with pneumonia in the livestock dense areas, reaching statistical significance in the year 2015. The same was observed for elderly with pneumonia (Table 5). Results remained robust after adjusting for SES (total sample: OR = 1.49, 99% CI 1.10–2.03; elderly group: OR = 1.53, 99% CI 1.10–2.10).

## 4. Discussion

Comparison between the study and control group demonstrated a significantly higher prevalence of pneumonia, lower respiratory tract infections, and acute respiratory symptoms in the livestock dense area. Subgroup analyses also showed significant differences in pneumonia among children and the elderly, as well as patients with COPD. Another interesting finding is that pneumonia patients in the livestock dense areas received broad-spectrum antibiotics more often compared to their counterparts in the control group (significantly in 2015), which might be a possible indication of the persistence or complications of respiratory health conditions. Similar findings were observed for the elderly subgroup with pneumonia. In addition, a higher prevalence of nonrespiratory health problems was observed, such as vertiginous syndrome and, to a lesser extent, depression. In general, there were no significant differences between areas with low and higher livestock density in chronic conditions such as asthma, COPD, or lung cancer.

Findings from the previous area comparisons over the period 2007–2013 [15] were verified to a great extent for the period 2014–2016. The fact that not all the recruited general practices and, therefore, registered patients/participants in the study and control area, were the same as in the earlier investigations in those areas adds to the robustness of these results. Nevertheless, contrary to the study of van Dijk et al. [15], we found no significant differences in the prevalence of chronic bronchitis/bronchiectasis for the years 2014–2016, despite the generally higher rates in the livestock dense areas. The current results are also consistent with analyses using individual estimates of exposure to different livestock types in relation to primary care-registered data, showing an increased prevalence of pneumonia with higher livestock density [9,14,16]. This was also the case for studies including self-reported outcome assessments [29,30], despite that prevalence estimates based on self-reported questionnaires can deviate from those extracted from medical records [31,32]. Moreover, although less research has focused on respiratory patients, the higher rates of respiratory symptoms and infections we found in COPD patients in the exposed areas seem to be in agreement with findings of earlier studies in the Netherlands [17,18].

With regards to the significantly higher rates of vertiginous syndrome (including labyrinthitis and Ménière’s disease) in the livestock dense areas, this was also the case in the years before 2014 [15], but a plausible etiological mechanism in relation to livestock is, to the best of our knowledge, unknown. The same holds for the higher depression rates; however, a study in Canada showed increased prevalence of depression in residents living in the proximity of a large swine farm [33]. Additionally, previous research suggested that perceived odor annoyance could be a possible determinant of symptomatology among residents living close to animal feeding operations, including psychological symptoms such as anxiousness and sadness [34]. In the present study, gastrointestinal conditions such as gastroenteritis and chronic enteritis also appeared to be increased among residents in the livestock dense areas but not consistently different compared to the control areas. Increased gastrointestinal symptoms/infections have been previously documented in relation to high farm density [35,36].

There is currently no clear explanation for the underlying causative agent responsible for the consistent pneumonia risk. A potential pathway is that people become more susceptible to respiratory infections when exposed to substances such as mold, fine dust, endotoxins, and ammonia [21,37,38]. Taking previous findings into account in terms of consistency over the years, goat farms seem to be the source primarily associated with respiratory problems in the investigated areas in the Netherlands and, to a lesser extent, other livestock such as poultry [4,21,39]. Evidence suggests that prior infection with *Coxiella burnetii*, which is mostly found in goat farms and was the causative agent of the Q-fever epidemic in the Netherlands [13], could be a contributing factor to susceptibility to other infections [40,41,42]. However, the incidence of Q-fever in the last few years has dropped to pre-epidemic levels [21], and serological analyses in a subgroup of residents in the livestock dense areas showed no significant association between being seropositive for *Coxiella burnetii* and pneumonia history [29,30]. Although the Q-fever outbreak may have resulted in increased perceived risks of living close to goat farms, associations with pneumonia were not biased by residents who attributed their symptoms to farm proximity [43]. Investigation of causal pathogens on the basis of serological analyses may shed further light into the causal mechanisms that lead to respiratory effects.

We conducted a large epidemiological study in terms of sample size, range of examined health outcomes, and exploration of various subgroups of patients with respiratory problems, as well as susceptible age subgroups. Important strengths are also the use of diagnosed health outcomes registered in general practices, which reduces the risk for selection bias and outcome misclassification. Among the study limitations is that individual estimates of livestock exposures and emissions of causative pathogens were not included in this study. However, livestock farms in the current livestock dense areas were found to be an important source of endotoxins [22]. Additionally, only limited information about possible confounders was available in the EHRs, and therefore, we could only adjust for age, gender, and registry duration. Nevertheless, the potential confounding effect of SES indicators was evaluated in sensitivity analyses; since this did not alter the results, only results without adjustment for SES were displayed in the tables to enhance comparability with earlier epidemiological studies that performed similar analyses [15]. This is also in agreement with earlier research that has shown that adjustment for socioeconomic status did not change the associations between livestock exposure and health outcomes [44]. Data on occupational status was also not available. However, this concerns a small fraction of the study population, and exclusion of residents living or working on a livestock farm did not change findings in previous investigations in the same exposed areas [15,17]. Finally, a large number of statistical analyses was performed, which can increase the chance for false positives, to some extent. However, the fact that the main findings are in line with previous studies in the same exposed areas makes this unlikely, at least for the most consistently observed results. To deal with multiple testing, we also employed a conservative level of statistical significance and analyzed each year separately.

## 5. Conclusions

Prevalence of pneumonia, lower respiratory tract infections, and respiratory symptoms was significantly and consistently higher in the areas with high livestock density. The present results are generally in agreement with prior findings in the same livestock dense areas. Identification of causal pathogens on the basis of serological analyses could further elucidate the underlying mechanisms behind the observed health effects.

## Figures and Tables

**Table 1:** Differences (OR, 99% CI) ^a^ per year in various acute and chronic conditions between the study and control areas, based on the total sample (statistically significant results in bold) *.

	2014	2015	2016
Pneumonia	**1.45 (1.00–2.10)**	**1.58 (1.09–2.30)**	**1.60 (1.13–2.28)**
Lower respiratory tract infections	1.31 (0.94–1.83)	**1.44 (1.01–2.05)**	**1.46 (1.03–2.05)**
Hay fever/Allergic rhinitis	0.95 (0.75–1.21)	1.00 (0.80–1.25)	1.01 (0.78–1.30)
Asthma ^b^	0.87 (0.68–1.12)	0.84 (0.66–1.06)	0.85 (0.68–1.07)
Cough, shortness of breath/dyspnea, wheezing	1.14 (0.90–1.45)	**1.27 (1.03–1.57)**	1.27 (0.98–1.66)
Chronic bronchitis/bronchiectasis ^c^	1.38 (0.83–2.29)	1.42 (0.87–2.33)	1.45 (0.93–2.27)
COPD ^c^	0.97 (0.77–1.22)	0.98 (0.78–1.24)	1.01 (0.81–1.25)
Lung cancer ^c^	1.09 (0.89–1.33)	1.09 (0.89–1.35)	1.28 (0.92–1.78)
Upper respiratory tract infections	0.93 (0.76–1.14)	0.95 (0.77–1.16)	0.98 (0.76–1.25)
Influenza	1.19 (0.59–2.38)	1.03 (0.50–2.15)	1.15 (0.76–1.74)
Chronic enteritis/Ulcerative colitis	1.13 (0.92–1.40)	1.13 (0.90–1.42)	1.15 (0.91–1.44)
Vertigo/Dizziness	1.18 (0.86–1.63)	1.12 (0.84–1.50)	1.16 (0.83–1.62)
Eczema	1.11 (0.72–1.70)	1.12 (0.75–1.67)	1.07 (0.72–1.59)
Gastroenteritis	1.08 (0.80–1.46)	1.14 (0.84–1.54)	**1.42 (1.02–1.98)**
Coronary heart disease ^c^	1.12 (0.91–1.37)	1.07 (0.88–1.31)	1.08 (0.90–1.29)
Vertiginous syndrome	**1.44 (1.12–1.85)**	**1.45 (1.10–1.92)**	**1.40 (1.06–1.84)**
Depression	1.25 (0.97–1.60)	**1.26 (1.01–1.58)**	1.23 (0.98–1.55)
Hypertension ^c^	0.99 (0.82–1.20)	0.98 (0.82–1.19)	0.98 (0.81–1.18)

^a^ Adjusted for age, gender, and part of the year during which a patient was registered at a general practice. ^b^ Age ≥ 6 years. ^c^ Age ≥ 40 years. Abbreviations: OR, odds ratio; CI, confidence interval; and COPD, chronic obstructive pulmonary disease. * *p* < 0.01.

**Table 2:** Differences (OR, 99% CI) ^a^ per year in various comorbid conditions between livestock dense areas and control areas among patients with pneumonia (total sample) (statistically significant results in bold) *.

	2014	2015	2016
Hay fever/Allergic rhinitis	0.95 (0.54–1.66)	0.89 (0.54–1.49)	0.83 (0.47–1.47)
Asthma ^b^	**0.65 (0.46–0.93)**	0.68 (0.46–1.01)	0.80 (0.55–1.17)
Cough, shortness of breath/dyspnea, wheezing	1.35 (0.82–2.23)	1.26 (0.80–2.01)	1.45 (0.81–2.60)
Chronic bronchitis/bronchiectasis ^c^	1.44 (0.74–2.81)	1.64 (0.75–3.57)	1.07 (0.57–2.02)
COPD ^c^	0.95 (0.63–1.43)	0.94 (0.64–1.38)	0.83 (0.62–1.13)
Lung cancer ^c^	1.19 (0.62–2.28)	0.84 (0.46–1.55)	1.25 (0.62–2.50)
Upper respiratory tract infections	0.90 (0.65–1.25)	**0.77 (0.61–0.99)**	0.85 (0.64–1.13)
Influenza	2.74 (0.75–10.0)	0.84 (0.26–2.78)	1.06 (0.57–1.96)
Chronic enteritis/Ulcerative colitis	1.08 (0.38–3.04)	1.10 (0.41–2.97)	1.35 (0.55–3.34)
Vertigo/Dizziness	1.44 (0.78–2.68)	1.27 (0.65–2.45)	1.19 (0.64–2.21)
Eczema	1.22 (0.70–2.12)	0.87 (0.53–1.43)	0.96 (0.57–1.61)
Gastroenteritis	1.44 (0.73–2.86)	0.83 (0.43–1.59)	1.66 (0.82–3.35)
Coronary heart disease ^c^	1.33 (0.96–1.85)	0.98 (0.74–1.29)	0.96 (0.65–1.40)
Vertiginous syndrome	1.91 (0.84–4.32)	1.93 (0.84–4.43)	1.15 (0.52–2.52)
Depression	1.24 (0.71–2.16)	1.18 (0.71–1.97)	1.30 (0.81–2.07)
Hypertension ^c^	0.93 (0.67–1.29)	1.00 (0.74–1.34)	1.08 (0.78–1.50)

^a^ Adjusted for age, gender, and part of the year during which a patient was registered at a general practice. ^b^ Age ≥ 6 years. ^c^ Age ≥ 40 years. Abbreviations: OR, odds ratio and CI, confidence interval. * *p* < 0.01.

**Table 3:** Differences (OR, 99% CI) ^a^ per year in various comorbid conditions between livestock dense areas and control areas among asthma patients ^b^ without comorbid COPD.

	2014	2015	2016
Pneumonia	1.00 (0.65–1.55)	1.21 (0.72–2.04)	1.42 (0.88–2.27)
Lower respiratory tract infections	1.00 (0.70–1.44)	1.19 (0.73–1.93)	1.39 (0.88–2.19)
Hay fever/Allergic rhinitis	0.96 (0.68–1.34)	1.01 (0.73–1.40)	1.04 (0.75–1.44)
Cough, shortness of breath/dyspnea, wheezing	1.27 (0.86–1.88)	1.33 (0.92–1.91)	1.45 (1.00–2.10) ^‡^
Lung cancer ^c^	1.03 (0.37–2.86)	0.60 (0.20–1.81)	0.86 (0.30–2.45)
Upper respiratory tract infections	0.93 (0.74–1.17)	0.92 (0.74–1.14)	0.88 (0.64–1.19)
Influenza	0.86 (0.27–2.74)	0.95 (0.25–3.55)	1.01 (0.56–1.82)
Chronic enteritis/Ulcerative colitis	1.09 (0.69–1.72)	1.00 (0.63–1.58)	0.99 (0.63–1.56)
Vertigo/Dizziness	1.28 (0.82–1.98)	1.17 (0.78–1.75)	1.42 (0.91–2.20)
Eczema	1.04 (0.71–1.50)	1.07 (0.73–1.56)	1.11 (0.78–1.60)
Gastroenteritis	0.89 (0.53–1.50)	1.18 (0.73–1.91)	1.54 (0.93–2.55)
Coronary heart disease ^c^	1.01 (0.72–1.40)	0.97 (0.69–1.37)	0.98 (0.71–1.36)
Vertiginous syndrome	1.20 (0.79–1.82)	1.40 (0.89–2.20)	1.16 (0.84–1.59)
Depression	1.23 (0.80–1.87)	1.35 (0.95–1.92)	1.16 (0.73–1.85)
Hypertension ^c^	1.04 (0.81–1.34)	1.08 (0.83–1.39)	1.08 (0.83–1.41)

^a^ Adjusted for age, gender, and part of the year during which a patient was registered at a general practice. ^b^ Age ≥ 6 years. ^c^ Age ≥ 40 years. Abbreviations: OR, odds ratio and CI, confidence interval. ^‡^ *p* = 0.01.

**Table 4:** Differences (OR, 99% CI) ^a^ per year in various comorbid conditions between livestock dense areas and control areas among COPD patients ^b^ without comorbid asthma (statistically significant results in bold) *.

	2014	2015	2016
Pneumonia	**1.70 (1.03–2.79)**	**1.70 (1.07–2.71)**	1.51 (0.99–2.28)
Lower respiratory tract infections	**1.58 (1.01–2.47)**	**1.61 (1.04–2.49)**	1.46 (0.99–2.16)
Hay fever/Allergic rhinitis	0.63 (0.39–1.01)	0.75 (0.44–1.26)	0.93 (0.52–1.65)
Cough, shortness of breath/dyspnea, wheezing	1.36 (0.84–2.19)	**1.60 (1.06–2.42)**	1.55 (0.97–2.49)
Lung cancer ^c^	1.27 (0.85–1.91)	1.18 (0.78–1.78)	1.17 (0.76–1.82)
Upper respiratory tract infections	0.97 (0.74–1.28)	1.03 (0.72–1.46)	0.99 (0.69–1.41)
Influenza	0.62 (0.15–2.50)	0.63 (0.13–3.09)	1.17 (0.56–2.45)
Chronic enteritis/Ulcerative colitis	1.09 (0.55–2.17)	1.03 (0.49–2.20)	1.11 (0.56–2.20)
Vertigo/Dizziness	1.39 (0.85–2.24)	1.23 (0.64–2.37)	1.32 (0.77–2.26)
Eczema	0.95 (0.53–1.70)	1.00 (0.55–1.81)	1.07 (0.58–2.00)
Gastroenteritis	0.98 (0.53–1.81)	0.98 (0.53–1.81)	0.78 (0.39–1.55)
Coronary heart disease ^c^	1.15 (0.91–1.46)	1.10 (0.88–1.37)	1.19 (0.95–1.48)
Vertiginous syndrome	**1.63 (1.03–2.6)**	**1.94 (1.07–3.52)**	1.69 (0.91–3.15)
Depression	**1.62 (1.11–2.35)**	**1.65 (1.14–2.39)**	1.37 (0.89–2.11)
Hypertension ^c^	1.09 (0.82–1.46)	1.13 (0.85–1.50)	1.08 (0.80–1.46)

^a^ Adjusted for age, gender, and part of the year during which a patient was registered at a general practice. ^b^ Age ≥ 40 years. Abbreviations: OR, odds ratio and CI, confidence interval. * *p* < 0.01.

**Table 5:** Differences (OR, 99% CI) ^a^ per year in antibiotic prescriptions between the livestock dense areas and control areas (statistically significant results in bold) *.

	2014	2015	2016
**Broad-spectrum antibiotics ^÷^**			
Total sample	0.92 (0.75–1.13)	0.86 (0.68–1.08)	0.96 (0.75–1.24)
Children ^b^	1.03 (0.77–1.37)	0.93 (0.69–1.25)	0.97 (0.71–1.34)
Elderly ^c^	0.98 (0.80–1.20)	0.95 (0.77–1.17)	1.02 (0.81–1.29)
Patients with pneumonia (total)	1.23 (0.87–1.74)	**1.49 (1.10–2.03)**	1.27 (0.82–1.96)
Children with pneumonia ^b^	1.42 (0.74–2.71)	2.10 (0.85–5.20)	1.37 (0.51–3.68)
Elderly with pneumonia ^c^	1.43 (0.93–2.19)	**1.53 (1.10–2.14)**	1.41 (0.89–2.23)
All asthma patients (without comorbid COPD) ^d^	0.88 (0.67–1.14)	0.84 (0.62–1.12)	0.82 (0.60–1.13)
Children with asthma ^e^	1.26 (0.72–2.21)	1.24 (0.65–2.37)	0.67 (0.38–1.18)
Elderly with asthma ^c^	0.90 (0.55–1.46)	0.88 (0.61–1.26)	0.83 (0.54–1.25)
COPD patients (without comorbid asthma) ^f^	1.14 (0.91–1.42)	1.08 (0.83–1.41)	1.22 (0.92–1.60)
ACOS patients ^f^	1.23 (0.85–1.78)	0.93 (0.62–1.38)	0.94 (0.59–1.50)
**All anti-infectives ^L^**			
Total sample	0.94 (0.82–1.08)	0.89 (0.77–1.03)	0.98 (0.79–1.21)
Children ^b^	0.99 (0.78–1.27)	0.90 (0.69–1.18)	1.06 (0.81–1.37)
Elderly ^c^	0.94 (0.81–1.09)	0.88 (0.77–1.02)	0.99 (0.79–1.23)
Patients with pneumonia (total)	0.92 (0.61–1.39)	0.98 (0.72–1.34)	0.92 (0.60–1.41)
Children with pneumonia ^b^	0.92 (0.43–1.99)	0.99 (0.43–2.25)	1.39 (0.50–3.90)
Elderly with pneumonia ^c^	1.03 (0.63–1.67)	1.03 (0.71–1.50)	0.91 (0.55–1.49)
All asthma patients (without comorbid COPD) ^d^	0.91 (0.74–1.12)	0.88 (0.70–1.11)	0.88 (0.70–1.10)
Children with asthma ^e^	0.97 (0.65–1.45)	0.92 (0.54–1.54)	0.88 (0.54–1.43)
Elderly with asthma ^c^	0.93 (0.65–1.32)	0.83 (0.59–1.18)	0.81 (0.61–1.07)
All COPD patients (without comorbid asthma) ^f^	1.00 (0.83–1.21)	0.94 (0.76–1.16)	1.01 (0.76–1.36)
ACOS patients ^f^	1.12 (0.78–1.62)	0.95 (0.64–1.40)	0.96 (0.57–1.63)

^a^ Adjusted for age, gender, and part of the year during which a patient was registered at a general practice. ^b^ Age 0–14 years. ^c^ Age ≥ 65 years, without comorbid COPD. ^d^ Age ≥ 6 years. ^e^ Age 6–14 years, without comorbid chronic bronchitis. ^f^ Age ≥ 40 years. Abbreviations: OR, odds ratio; CI, confidence interval; and ACOS, asthma and COPD overlap syndrome. * *p* < 0.01 ^÷^ anatomical therapeutic chemical (ATC) codes “J01CA04” (amoxicillin) + “J01AA02” (doxycycline) and ^L^ ATC group “J” (anti-infectives for systemic use).

## Appendix A

Overview of the Total Sample and Definition of the Different Patient Subgroups in the Present Analyses

**Table A1:** Overview of the total sample in the livestock dense areas and control areas.

	Selected Areas			
	Rural Areas with High Livestock Density (Livestock Dense Areas)		Rural Areas with Low Livestock Density (Control Areas)	
	General Practices	Patients	General Practices	Patients
2014	25	106,688	22	75,390
2015	24	103,621	22	74,746
2016	24	102,975	22	76,704

**Table A2:** Definition of the different patient subgroups in the present analyses.

Subgroup	Definition
**Susceptible age subgroups**	
Children	Age 0–14 years
Elderly	Age ≥ 65 years
**Respiratory patient groups**	
Pneumonia	Every patient registered with the ICPC code R81
Asthma	Every patient of age ≥ 6 years without COPD, registered with the ICPC code R96
COPD	Every patient of age ≥ 40 years without asthma, registered with the ICPC code R91 or R95
ACOS	Every patient of age ≥ 40 years diagnosed with COPD (ICPC codes R91 or R95) and asthma (ICPC code R96)
**Respiratory patient groups (alternative case definitions)**	
Asthma	Every patient registered with the ICPC code R96
COPD	Every patient of age ≥ 40 years registered with the ICPC code R91 or R95
ACOS	Every patient of age ≥ 40 years diagnosed with COPD (ICPC codes R91 or R95) or asthma (ICPC code R96)

ICPC, international classification of primary care; COPD, chronic obstructive pulmonary disease; and ACOS, asthma and COPD overlap syndrome.

Overview of the Included Health Outcomes with the Corresponding Registration Codes in Primary Care

**Table A3:** Overview of the included health outcomes with the corresponding registration codes in primary care.

Health Symptoms/Conditions	ICPC Code (s)
Pneumonia	R81
Lower respiratory tract infections	R81–R83
Hay fever/Allergic rhinitis	R97
Asthma	R96
Cough, shortness of breath/dyspnea, wheezing	R02, R03, R05
Chronic bronchitis/ bronchiectasis	R91
COPD	R91, R95
Lung cancer	R84–R85
Upper respiratory tract infections	R74–R78
Influenza	R80
Chronic enteritis/Ulcerative colitis	D94
Vertigo/Dizziness	N17
Eczema	S87
Gastroenteritis	D70, D73
Coronary heart disease	K74–K76
Vertiginous syndrome	H82
Depression	P03, P76
Hypertension	K86

Sample Characteristics

**Table A4:** Patient characteristics of the population in the study and control areas.

	2014	2015	2016
**Livestock dense areas**			
Gender (% female)	49.6	49.5	49.5
Age (mean (SD)	42.6 (23.1)	43.1 (23.2)	43.6 (23.3)
Children n	15,674	14,795	14,108
Elderly n	21,280	21,528	22,178
*Respiratory patient groups*			
**Pneumonia**			
Subjects n	2865	3349	3032
Gender (% female)	50.0	47.0	47.5
Age (mean (SD)	56.9 (26.4)	57.8 (26.3)	57.6
**Asthma**			
Subjects n	6933	6824	7063
Gender (% female)	50.5	50.2	50.2
Age (mean (SD)	37.8 (21.2)	38.3 (21.4)	38.9 (21.5)
**COPD**			
Subjects n	2814	2768	2738
Gender (% female)	43.9	44.3	44.7
Age (mean (SD)	69.5 (11.1)	69.7 (11.1)	69.8 (11.1)
**ACOS**			
Subjects n	945	950	981
Gender (% female)	52.6	52.5	51.9
Age (mean (SD)	66.4 (11.7)	67.0 (11.7)	67.6 (11.7)
**CONTROL AREAS**			
*Total sample*			
Gender (% female)	49.9	49.7	49.7
Age (mean (SD)	43.0 (23.5)	43.5 (23.5)	43.6 (23.6)
Children n	11,526	11,080	11,118
Elderly n	15,991	16,211	16,999
*Respiratory patient groups*			
**Pneumonia**			
Subjects n	984	1107	1012
Gender (% female)	50.0	47.2	46.5
Age (mean (SD)	57.7 (26.6)	58.8 (25.7)	57.6
**Asthma**			
Subjects n	5597	5746	6011
Gender (% female)	53.2	53.2	52.9
Age (mean (SD)	39.5 (21.2)	40.0 (21.3)	40.6 (21.4)
**COPD**			
Subjects n	2205	2139	2184
Gender (% female)	43.5	43.9	44.0
Age (mean (SD)	70.7 (11.6)	71.1 (11.5)	71.1 (11.4)
**ACOS**			
Subjects n	738	749	773
Gender (% female)	53.9	54.1	53.8
Age (mean (SD)	66.1 (12.0)	66.5 (12.1)	67.0 (12.1)

**Table A5:** Average prevalence (%) of health conditions in the study and control area for the period 2014–2016.

	Average Prevalence 2014–2016	
	Livestock Dense Area	Control Area
Pneumonia	2.01	1.43
Lower respiratory tract infections	2.28	1.85
Hay fever/Allergic rhinitis	3.89	4.14
Asthma ^a^	8.14	9.55
Cough, shortness of breath/dyspnea, wheezing	7.68	6.61
Chronic bronchitis/bronchiectasis ^b^	1.59	1.25
COPD ^b^	6.35	6.99
Lung cancer ^b^	0.72	0.69
Upper respiratory tract infections	9.57	10.5
Influenza	0.62	0.76
Chronic enteritis/Ulcerative colitis	0.88	0.81
Vertigo/Dizziness	1.73	1.51
Eczema	9.54	8.87
Gastroenteritis	1.31	1.16
Coronary heart disease ^b^	8.99	8.81
Vertiginous syndrome	1.48	1.14
Depression	3.93	3.29
Hypertension ^b^	25.9	27.2

^a^ Age ≥ 6 years. ^b^ Age ≥ 40 years.

Results of Additional Analyses Based on Susceptible Age Subgroups

**Table A6:** Differences (OR, 99% CI) ^a^ per year in various comorbid conditions between livestock dense areas and control areas among children ^b^ (statistically significant results in bold) *

	2014	2015	2016
Pneumonia	1.43 (0.75–2.73)	1.98 (0.95–4.14)	**2.22 (1.20–4.1)**
Lower respiratory tract infections	1.31 (0.71–2.42)	1.80 (0.89–3.63)	1.87 (0.99–3.53)
Hay fever/Allergic rhinitis	1.01 (0.74–1.37)	1.06 (0.78–1.44)	1.07 (0.78–1.48)
Asthma ^c^	1.05 (0.74–1.48)	0.96 (0.69–1.35)	1.01 (0.70–1.46)
Cough, shortness of breath/dyspnea, wheezing	1.15 (0.82–1.61)	1.31 (0.96–1.78)	1.15 (0.83–1.58)
Upper respiratory tract infections	0.99 (0.78–1.25)	1.02 (0.82–1.26)	1.13 (0.81–1.58)
Influenza	1.19 (0.41–3.47)	0.79 (0.31–2.04)	1.23 (0.61–2.49)
Chronic enteritis/Ulcerative colitis	1.12 (0.20–6.35)	2.27 (0.28–18.6)	2.31 (0.24–21.8)
Vertigo/Dizziness	0.92 (0.41–2.03)	2.15 (0.83–5.57)	1.00 (0.38–2.60)
Eczema	1.11 (0.79–1.56)	1.14 (0.84–1.55)	1.09 (0.81–1.46)
Gastroenteritis	1.05 (0.74–1.48)	1.17 (0.83–1.64)	1.26 (0.88–1.82)
Vertiginous syndrome	1.77 (0.45–6.97)	3.68 (0.44–30.3)	10.9 (0.67–178.5)
Depression	0.70 (0.35–1.41)	0.84 (0.40–1.74)	0.80 (0.35–1.80)

^a^ Adjusted for age, gender, and part of the year during which a patient was registered at a general practice. ^b^ Age 0–4 years. ^c^ Age ≥ 6 years. Abbreviations: OR, odds ratio; CI, confidence interval; and i.n.c: insufficient number of cases. * *p* < 0.01.

**Table A7:** Differences (OR, 99% CI) ^a^ per year in various comorbid conditions between livestock dense areas and control areas among elderly ^b^ (statistically significant results in bold) *.

	2014	2015	2016
Pneumonia	1.49 (0.98–2.25)	**1.55 (1.09–2.20)**	**1.70 (1.18–2.45)**
Lower respiratory tract infections	1.38 (0.93–2.04)	**1.49 (1.06–2.10)**	**1.61 (1.13–2.29)**
Hay fever/Allergic rhinitis	0.91 (0.62–1.34)	0.97 (0.67–1.40)	0.99 (0.71–1.39)
Asthma	0.85 (0.64–1.14)	0.82 (0.62–1.08)	0.86 (0.67–1.11)
Cough, shortness of breath/dyspnea, wheezing	1.23 (0.94–1.62)	**1.33 (1.04–1.68)**	**1.38 (1.03–1.84)**
Chronic bronchitis/bronchiectasis	1.40 (0.85–2.30)	1.35 (0.82–2.22)	1.42 (0.90–2.24)
COPD	0.93 (0.76–1.15)	0.95 (0.77–1.17)	0.97 (0.80–1.18)
Lung cancer	1.07 (0.85–1.35)	1.04 (0.83–1.32)	1.23 (0.91–1.67)
Upper respiratory tract infections	0.93 (0.74–1.17)	0.92 (0.73–1.17)	0.85 (0.65–1.11)
Influenza	1.35 (0.55–3.29)	1.26 (0.46–3.46)	1.27 (0.77–2.08)
Chronic enteritis/Ulcerative colitis	1.11 (0.81–1.52)	1.16 (0.86–1.55)	1.15 (0.86–1.53)
Vertigo/Dizziness	1.28 (0.94–1.75)	1.27 (0.93–1.73)	1.16 (0.81–1.67)
Eczema	1.12 (0.62–2.04)	1.12 (0.64–1.97)	1.11 (0.63–1.95)
Gastroenteritis	1.02 (0.74–1.41)	1.02 (0.70–1.49)	1.31 (0.88–1.95)
Coronary heart disease	1.13 (0.92–1.40)	1.11 (0.90–1.37)	1.12 (0.92–1.35)
Vertiginous syndrome	**1.52 (1.11–2.08)**	**1.54 (1.11–2.14)**	**1.46 (1.05–2.03)**
Depression	**1.38 (1.02–1.86)**	**1.45 (1.08–1.94)**	1.30 (0.98–1.72)
Hypertension	1.00 (0.79–1.25)	1.00 (0.80–1.24)	1.00 (0.80–1.25)

^a^ Adjusted for age, gender, and part of the year during which a patient was registered at a general practice. ^b^ Age ≥ 65 years. Abbreviations: OR, odds ratio and CI, confidence interval. * *p* < 0.01.

Results of Additional Analyses Based on Age Subgroups of Respiratory Patients

**Table A8:** Differences (OR, 99% CI) ^a^ per year in various comorbid conditions between livestock dense areas and control areas among children ^b^ with pneumonia

	2014	2015	2016
Hay fever/Allergic rhinitis	0.73 (0.19–2.77)	0.30 (0.10–1.23)	0.80 (0.18–3.55)
Asthma ^c^	0.61 (0.20–1.89)	0.51 (0.20–1.3)	1.23 (0.36–4.13)
Cough, shortness of breath/dyspnea, wheezing	2.19 (0.98–4.85)	1.71 (0.82–3.57)	2.01 (0.81–4.96)
Upper respiratory tract infections	0.85 (0.46–1.54)	0.96 (0.51–1.81)	0.98 (0.42–2.29)
Influenza	i.n.c	i.n.c	1.83 (0.20–16.5)
Chronic enteritis/Ulcerative colitis	i.n.c	i.n.c	i.n.c
Vertigo/Dizziness	i.n.c	i.n.c	i.n.c
Eczema	1.37 (0.69–2.72)	0.76 (0.34–1.70)	0.85 (0.38–1.88)
Gastroenteritis	2.74 (0.64–11.8)	0.82 (0.30–2.27)	1.78 (0.33–9.69)
Vertiginous syndrome	i.n.c	i.n.c	i.n.c
Depression	i.n.c	i.n.c	i.n.c

^a^ Adjusted for age, gender, and part of the year during which a patient was registered at a general practice. ^b^ Age 0–14 years. ^c^ Age ≥ 6 years. Abbreviations: OR, odds ratio; CI, confidence interval; and i.n.c: insufficient number of cases. * *p* < 0.01.

**Table A9:** Differences (OR, 99% CI) ^a^ per year in various comorbid conditions between livestock dense areas and control areas among elderly patients with pneumonia ^b^ (statistically significant results in bold) *.

	2014	2015	2016
Hay fever/Allergic rhinitis	1.21 (0.40–3.60)	0.82 (0.35–1.94)	0.98 (0.37–2.55)
Asthma	0.58 (0.32–1.04)	0.73 (0.42–1.26)	0.77 (0.45–1.31)
Cough, shortness of breath/dyspnea, wheezing	1.27 (0.73–2.22)	1.36 (0.80–2.31)	1.19 (0.66–2.15)
Chronic bronchitis/bronchiectasis	1.48 (0.68–3.18)	1.64 (0.73–3.68)	1.01 (0.49–2.08)
COPD	0.89 (0.57–1.38)	0.87 (0.56–1.35)	0.84 (0.62–1.15)
Lung cancer	1.26 (0.61–2.62)	0.80 (0.40–1.60)	1.14 (0.51–2.54)
Upper respiratory tract infections	0.95 (0.64–1.39)	**0.71 (0.53–0.96)**	0.74 (0.50–1.09)
Influenza	2.97 (0.39–22.5)	0.91 (0.19–4.32)	1.15 (0.43–3.11)
Chronic enteritis/Ulcerative colitis	2.66 (0.35–20.3)	0.93 (0.21–4.01)	1.58 (0.46–5.38)
Vertigo/Dizziness	1.59 (0.77–3.30)	1.42 (0.61–3.28)	1.30 (0.63–2.66)
Eczema	1.34 (0.58–3.06)	0.93 (0.49–1.78)	1.22 (0.60–2.46)
Gastroenteritis	1.24 (0.54–2.82)	0.72 (0.30–1.69)	1.68 (0.62–4.57)
Coronary heart disease	**1.40 (1.00–1.96)**	0.98 (0.72–1.33)	0.93 (0.63–1.38)
Vertiginous syndrome	2.01 (0.76–5.32)	1.63 (0.66–4.03)	0.90 (0.36–2.26)
Depression	1.34 (0.69–2.59)	1.58 (0.81–3.07)	1.28 (0.64–2.56)
Hypertension	0.88 (0.57–1.37)	1.02 (0.69–1.50)	1.23 (0.81–1.86)

^a^ Adjusted for age, gender, and part of the year during which a patient was registered at a general practice. ^b^ Age ≥ 65 years. Abbreviations: OR, odds ratio and CI, confidence interval. * *p* < 0.01.

**Table A10:** Differences (OR, 99% CI) ^a^ per year in various comorbid conditions between livestock dense areas and control areas among children with asthma ^b^.

	2014	2015	2016
Pneumonia	0.95 (0.37–2.42)	0.91 (0.33–2.52)	1.73 (0.70–4.26)
Lower respiratory tract infections	1.04 (0.43–2.53)	0.92 (0.33–2.60)	1.38 (0.60–3.18)
Hay fever/Allergic rhinitis	0.79 (0.48–1.3)	0.90 (0.55–1.45)	0.81 (0.48–1.37)
Cough, shortness of breath/dyspnea, wheezing	1.56 (0.82–2.97)	1.30 (0.7–2.43)	1.26 (0.72–2.19)
Upper respiratory tract infections	1.01 (0.68–1.53)	0.90 (0.63–1.30)	0.77 (0.45–1.31)
Influenza	i.n.c	1.11 (0.15–8.32)	0.85 (0.30–2.43)
Chronic enteritis/Ulcerative colitis	i.n.c	2.93 (0.13–63.3)	i.n.c
Vertigo/Dizziness	i.n.c	2.00 (0.23–17.5)	0.32 (0.03–3.23)
Eczema	0.93 (0.63–1.36)	0.98 (0.67–1.43)	1.00 (0.67–1.49)
Gastroenteritis	0.64 (0.26–1.56)	1.07 (0.36–3.17)	1.38 (0.47–4.05)
Vertiginous syndrome	i.n.c	i.n.c	i.n.c
Depression	0.69 (0.13–3.57)	1.33 (0.19–9.30)	0.80 (0.03–17.7)

^a^ Adjusted for age, gender, and part of the year during which a patient was registered at a general practice. ^b^ Age 6–14 years, without comorbid chronic bronchitis. Abbreviations: OR, odds ratio; CI, confidence interval; and i.n.c: insufficient number of cases.

**Table A11:** Differences (OR, 99% CI) ^a^ per year in various comorbid conditions between livestock dense areas and control areas among elderly asthma patients ^b^, without comorbid COPD.

	2014	2015	2016
Pneumonia	1.18 (0.57–2.42)	1.73 (0.81–3.66)	1.82 (0.84–3.95)
Lower respiratory tract infections	1.19 (0.58–2.45)	1.86 (0.96–3.61)	2.09 (1.00–4.36) ^‡^
Hay fever/Allergic rhinitis	0.67 (0.34–1.33)	0.77 (0.43–1.38)	1.20 (0.60–2.41)
Cough, shortness of breath/dyspnea, wheezing	1.39 (0.82–2.38)	1.46 (0.88–2.44)	1.40 (0.85–2.31)
Lung cancer	1.03 (0.31–3.40)	0.69 (0.19–2.51)	1.10 (0.34–3.55)
Upper respiratory tract infections	1.02 (0.63–1.62)	0.91 (0.61–1.38)	0.88 (0.52–1.48)
Influenza	0.66 (0.06–7.02)	1.30 (0.13–12.8)	1.56 (0.38–6.40)
Chronic enteritis/Ulcerative colitis	3.09 (0.82–11.6)	3.00 (0.79–11.4)	1.55 (0.51–4.72)
Vertigo/Dizziness	1.87 (0.85–4.10)	1.19 (0.66–2.14)	1.48 (0.78–2.77)
Eczema	1.45 (0.67–3.10)	1.52 (0.80–2.88)	1.57 (0.82–3.02)
Gastroenteritis	0.30 (0.08–1.09)	0.94 (0.36–2.44)	1.32 (0.37–4.68)
Coronary heart disease	1.10 (0.72–1.68)	1.10 (0.71–1.71)	1.08 (0.73–1.60)
Vertiginous syndrome	0.87 (0.44–1.73)	1.10 (0.53–2.27)	1.08 (0.58–2.01)
Depression	1.28 (0.66–2.51)	1.33 (0.77–2.29)	1.15 (0.65–2.03)
Hypertension	0.96 (0.70–1.32)	0.96 (0.70–1.33)	1.07 (0.79–1.44)

^a^ Adjusted for age, gender, and part of the year during which a patient was registered at a general practice. Abbreviations: OR, odds ratio and CI, confidence interval. ^b^ Age ≥ 65 years. ^‡^ *p* = 0.01.

Results of All Analyses Based on Alternative Case Definitions for Respiratory Patient Groups

**Table A12:** Differences (OR, 99% CI) ^a^ per year between livestock dense areas and control areas among asthma patients ^b^ (statistically significant results in bold) *.

	2014	2015	2016
Pneumonia	1.10 (0.72–1.66)	1.25 (0.75–2.08)	1.38 (0.88–2.17)
Lower respiratory tract infections	1.06 (0.74–1.51)	1.23 (0.77–1.96)	1.36 (0.88–2.10)
Hay fever/Allergic rhinitis	0.97 (0.70–1.36)	1.02 (0.75–1.40)	1.06 (0.77–1.45)
Cough, shortness of breath/dyspnea, wheezing	1.28 (0.86–1.89)	1.37 (0.96–1.96)	**1.53 (1.05–2.24)**
Chronic bronchitis/bronchiectasis ^c^	1.79 (0.90–3.56)	1.87 (1.00–3.49) ^‡^	1.84 (1.00–3.37) ^‡^
COPD ^c^	1.15 (0.81–1.63)	1.18 (0.84–1.65)	1.21 (0.86–1.70)
Lung cancer ^c^	1.27 (0.62–2.60)	0.99 (0.48–2.02)	1.26 (0.65–2.47)
Upper respiratory tract infection	0.95 (0.74–1.22)	0.94 (0.74–1.19)	0.85 (0.62–1.16)
Influenza	1.18 (0.38–3.63)	0.88 (0.27–2.81)	1.00 (0.58–1.72)
Chronic enteritis/Ulcerative colitis	0.96 (0.63–1.45)	0.93 (0.61–1.41)	0.95 (0.63–1.43)
Vertigo/Dizziness	1.26 (0.82–1.94)	1.14 (0.78–1.67)	1.46 (0.99–2.15)
Eczema	1.06 (0.73–1.54)	1.08 (0.74–1.57)	1.12 (0.78–1.6)
Gastroenteritis	0.89 (0.55–1.43)	1.15 (0.75–1.76)	1.43 (0.93–2.2)
Coronary heart disease ^c^	0.98 (0.69–1.39)	0.95 (0.68–1.33)	0.94 (0.70–1.28)
Vertiginous syndrome	1.27 (0.89–1.81)	1.42 (0.99–2.05)	1.12 (0.77–1.63)
Depression	1.26 (0.85–1.86)	1.32 (0.95–1.84)	1.20 (0.89–1.62)
Hypertension ^c^	1.06 (0.83–1.37)	1.07 (0.84–1.38)	1.08 (0.84–1.38)
Broad-spectrum antibiotics ^÷^	0.95 (0.75–1.22)	0.87 (0.65–1.15)	0.87 (0.64–1.18)
All anti-infectives ^L^	0.96 (0.79–1.17)	0.90 (0.72–1.14)	0.93 (0.72–1.19)

^a^ Adjusted for age, gender, and part of the year during which a patient was registered at a general practice. ^b^ Age ≥ 6 years; patients with comorbid COPD were not excluded. ^c^ Age ≥ 40 years. Abbreviations: OR, odds ratio and CI, confidence interval. * *p* < 0.01. ^‡^ *p* = 0.01. ^÷^ ATC codes “J01CA04” (amoxicillin) + “J01AA02” (doxycycline). ^L^ ATC group “J” (anti-infectives for systemic use).

**Table A13:** Differences (OR, 99% CI) ^a^ per year between livestock dense areas and control areas among children with asthma ^b^.

	2014	2015	2016
Pneumonia	0.95 (0.37–2.41)	0.86 (0.32–2.32)	1.71 (0.70–4.18)
Lower respiratory tract infections	1.04 (0.42–2.52)	0.88 (0.32–2.40)	1.36 (0.59–3.14)
Hay fever/Allergic rhinitis	0.79 (0.48–1.29)	0.89 (0.55–1.44)	0.80 (0.48–1.32)
Cough, shortness of breath/dyspnea, wheezing	1.57 (0.83–2.99)	1.30 (0.70–2.42)	1.25 (0.72–2.17)
Upper respiratory tract infection	1.05 (0.69–1.58)	0.92 (0.64–1.32)	0.79 (0.46–1.35)
Influenza	i.n.c	1.11 (0.15–8.20)	0.84 (0.30–2.40)
Chronic enteritis/Ulcerative colitis	i.n.c	2.92 (0.13–63.3)	i.n.c
Vertigo/Dizziness	i.n.c	2.01 (0.23–17.5)	0.32 (0.03–3.24)
Eczema	0.93 (0.63–1.35)	0.98 (0.67–1.43)	0.99 (0.67–1.47)
Gastroenteritis	0.63 (0.26–1.55)	1.13 (0.38–3.38)	1.56 (0.53–4.62)
Vertiginous syndrome	i.n.c	i.n.c	i.n.c
Depression	0.69 (0.13–3.56)	1.32 (0.19–9.30)	0.8 (0.03–17.7)
Broad-spectrum antibiotics ^÷^	1.27 (0.73–2.21)	1.20 (0.63–2.29)	0.70 (0.40–1.23)
All anti-infectives ^L^	1.02 (0.69–1.51)	0.92 (0.55–1.55)	0.89 (0.55–1.45)

^a^ Adjusted for age, gender, and part of the year during which a patient was registered at a general practice. ^b^ Age 6–14 years; patients with comorbid chronic bronchitis were not excluded. Abbreviations: OR, odds ratio; CI, confidence interval; and i.n.c: insufficient number of cases. ^÷^ ATC codes “J01CA04” (amoxicillin) ” + “J01AA02” (doxycycline). ^L^ ATC group “J” (anti-infectives for systemic use).

**Table A14:** Differences (OR, 99% CI) ^a^ per year between livestock dense areas and control areas among elderly asthma patients ^b^ (statistically significant results in bold) *.

	2014	2015	2016
Pneumonia	1.09 (0.60–1.98)	1.38 (0.71–2.67)	1.55 (0.86–2.80)
Lower respiratory tract infections	1.00 (0.56–1.75)	1.41 (0.78–2.53)	1.68 (0.96–2.92)
Hay fever/Allergic rhinitis	0.71 (0.40–1.25)	0.78 (0.46–1.32)	1.15 (0.63–2.08)
Cough, shortness of breath/dyspnea, wheezing	1.36 (0.83–2.21)	1.42 (0.93–2.17)	**1.62 (1.01–2.60)**
Chronic bronchitis/bronchiectasis	1.74 (0.82–3.73)	1.85 (0.91–3.72)	1.76 (0.89–3.48)
COPD	1.16 (0.80–1.69)	1.19 (0.82–1.74)	1.24 (0.86–1.80)
Lung cancer	1.17 (0.51–2.70)	0.95 (0.40–2.26)	1.35 (0.58–3.15)
Upper respiratory tract infection	1.05 (0.68–1.62)	0.93 (0.63–1.37)	0.85 (0.50–1.43)
Influenza	0.73 (0.14–3.82)	1.09 (0.16–7.48)	1.22 (0.42–3.49)
Chronic enteritis/Ulcerative colitis	1.86 (0.66–5.22)	2.02 (0.64–6.35)	1.47 (0.54–4.00)
Vertigo/Dizziness	1.47 (0.82–2.61)	1.23 (0.76–1.99)	1.56 (0.93–2.62)
Eczema	1.48 (0.71–3.05)	1.42 (0.77–2.61)	1.44 (0.79–2.62)
Gastroenteritis	0.48 (0.18–1.26)	0.98 (0.44–2.20)	1.09 (0.49–2.40)
Coronary heart disease	1.01 (0.69–1.49)	1.01 (0.69–1.46)	1.00 (0.71–1.42)
Vertiginous syndrome	1.18 (0.68–2.05)	1.45 (0.81–2.58)	1.17 (0.70–1.98)
Depression	1.22 (0.69–2.17)	1.25 (0.83–1.89)	1.17 (0.77–1.79)
Hypertension	1.04 (0.76–1.42)	1.02 (0.75–1.39)	1.09 (0.82–1.45)
Broad-spectrum antibiotics ^÷^	1.00 (0.68–1.45)	0.96 (0.70–1.33)	0.92 (0.63–1.36)
All anti-infectives ^L^	0.96 (0.71–1.31)	0.89 (0.65–1.20)	0.94 (0.67–1.30)

^a^ Adjusted for age, gender, and part of the year during which a patient was registered at a general practice. ^b^ Age ≥65 years; patients with comorbid COPD were not excluded. Abbreviations: OR, odds ratio and CI, confidence interval. * *p* < 0.01. ^÷^ ATC codes “J01CA04" (amoxicillin) + “J01AA02” (doxycycline). ^L^ ATC group “J” (anti-infectives for systemic use).

**Table A15:** Differences (OR, 99% CI) ^a^ per year between livestock dense areas and control areas among COPD patients ^b^ (statistically significant results in bold) *.

	2014	2015	2016
Pneumonia	1.55 (0.98–2.44)	1.56 (0.99–2.47)	1.44 (0.94–2.22)
Asthma	0.96 (0.70–1.31)	0.92 (0.66–1.28)	0.95 (0.68–1.34)
Lower respiratory tract infections	1.44 (0.96–2.15)	1.52 (0.98–2.34)	1.41 (0.95–2.11)
Hay fever/Allergic rhinitis	0.79 (0.48–1.30)	0.89 (0.52–1.52)	1.16 (0.67–2.00)
Cough, shortness of breath/dyspnea, wheezing	1.36 (0.85–2.17)	**1.62 (1.08–2.41)**	**1.68 (1.07–2.65)**
Lung cancer	1.30 (0.91–1.88)	1.22 (0.84–1.76)	1.24 (0.82–1.88)
Upper respiratory tract infections	1.02 (0.77–1.36)	1.05 (0.74–1.47)	0.89 (0.63–1.26)
Influenza	1.01 (0.31–3.28)	0.69 (0.18–2.58)	1.28 (0.69–2.38)
Chronic enteritis/Ulcerative colitis	0.88 (0.52–1.49)	0.90 (0.51–1.61)	0.98 (0.57–1.68)
Vertigo/Dizziness	1.31 (0.85–2.00)	1.16 (0.65–2.04)	1.35 (0.84–2.16)
Eczema	1.05 (0.62–1.78)	1.04 (0.63–1.71)	1.11 (0.65–1.87)
Gastroenteritis	0.90 (0.51–1.57)	0.94 (0.53–1.66)	0.80 (0.42–1.53)
Coronary heart disease	1.08 (0.83–1.41)	1.03 (0.79–1.33)	1.09 (0.87–1.37)
Vertiginous syndrome	**1.59 (1.03–2.45)**	**1.88 (1.14–3.10)**	1.44 (0.88–2.36)
Depression	**1.53 (1.10–2.12)**	**1.48 (1.07–2.05)**	1.36 (0.95–1.94)
Hypertension	1.09 (0.82–1.43)	1.10 (0.83–1.44)	1.06 (0.80–1.40)
Broad-spectrum antibiotics ^÷^	1.15 (0.93–1.42)	1.05 (0.80–1.37)	1.14 (0.86–1.50)
All anti-infectives ^L^	1.03 (0.86–1.23)	0.95 (0.78–1.16)	1.02 (0.75–1.38)

^a^ Adjusted for age, gender, and part of the year during which a patient was registered at a general practice. ^b^ Age ≥40 years; patients with comorbid asthma were not excluded. Abbreviations: OR, odds ratio and CI, confidence interval. * *p* < 0.01. ^÷^ ATC codes “J01CA04” (amoxicillin) + “J01AA02” (doxycycline). ^L^ ATC group “J” (anti-infectives for systemic use).

**Table A16:** Differences (OR, 99% CI) ^a^ per year in various comorbid conditions between the livestock dense areas and control areas among ACOS patients ^b^ (statistically significant results in bold) *.

	2014	2015	2016
Pneumonia	1.24 (0.67–2.26)	1.20 (0.58–2.47)	1.20 (0.58–2.44)
Lower respiratory tract infections	1.12 (0.64–1.94)	1.21 (0.61–2.38)	1.18 (0.60–2.32)
Hay fever/Allergic rhinitis	1.12 (0.55–2.31)	1.15 (0.58–2.26)	1.47 (0.65–3.30)
Cough, shortness of breath/dyspnea, wheezing	1.27 (0.69–2.34)	1.58 (0.97–2.55)	**1.96 (1.14–3.38)**
Lung cancer	1.49 (0.54–4.10)	1.36 (0.51–3.66)	1.75 (0.67–4.57)
Upper respiratory tract infections	1.10 (0.68–1.78)	1.02 (0.61–1.72)	0.63 (0.37–1.07)
Influenza	1.60 (0.28–8.99)	0.49 (0.10–2.96)	1.65 (0.48–5.64)
Chronic enteritis/Ulcerative colitis	0.48 (0.17–1.37)	0.60 (0.21–1.73)	0.61 (0.22–1.70)
Vertigo/Dizziness	1.07 (0.50–2.29)	0.96 (0.50–1.85)	1.57 (0.81–3.04)
Eczema	1.27 (0.67–2.41)	1.15 (0.65–2.03)	1.14 (0.65–2.00)
Gastroenteritis	0.72 (0.26–1.96)	0.93 (0.36–2.42)	0.85 (0.27–2.67)
Coronary heart disease	0.85 (0.52–1.39)	0.82 (0.54–1.25)	0.82 (0.55–1.23)
Vertiginous syndrome	1.40 (0.65–3.04)	1.61 (0.71–3.66)	1.01 (0.45–2.25)
Depression	1.32 (0.76–2.31)	1.13 (0.70–1.83)	1.45 (0.86–2.44)
Hypertension	1.09 (0.73–1.62)	1.04 (0.71–1.53)	1.04 (0.75–1.43)

^a^ Adjusted for age, gender, and part of the year during which a patient was registered at a general practice. ^b^ Asthma and COPD overlap syndrome; age ≥40 years. Abbreviations: OR, odds ratio and CI, confidence interval. * *p* < 0.01.

**Table A17:** Differences (OR, 99% CI) a per year between livestock dense areas and control areas among ACOS patients (alternative definition) ^b^ (statistically significant results in bold) *.

	2014	2015	2016
Pneumonia	1.37 (0.90–2.08)	**1.56 (1.02–2.38)**	**1.54 (1.02–2.32)**
Lower respiratory tract infections	1.27 (0.89–1.82)	**1.54 (1.03–2.29)**	**1.51 (1.30–2.21)**
Hay fever/Allergic rhinitis	0.83 (0.55–1.25)	0.86 (0.58–1.29)	1.01 (0.68–1.50)
Cough, shortness of breath/dyspnea, wheezing	1.28 (0.82–1.98)	**1.48 (1.01–2.17)**	**1.61 (1.06–2.44)**
Lung cancer	1.29 (0.90–1.85)	1.17 (0.83–1.66)	1.24 (0.84–1.83)
Upper respiratory tract infections	0.96 (0.75–1.22)	0.96 (0.74–1.25)	0.86 (0.62–1.19)
Influenza	0.87 (0.34–2.28)	0.78 (0.26–2.34)	1.43 (0.87–2.34)
Chronic enteritis/Ulcerative colitis	1.02 (0.70–1.49)	0.98 (0.67–1.45)	1.01 (0.70–1.48)
Vertigo/Dizziness	1.39 (0.95–2.04)	1.21 (0.76–1.91)	1.45 (0.97–2.18)
Eczema	1.12 (0.72–1.76)	1.13 (0.72–1.77)	1.18 (0.75–1.87)
Gastroenteritis	0.88 (0.58–1.32)	0.97 (0.62–1.53)	1.01 (0.66–1.56)
Coronary heart disease	1.08 (0.84–1.41)	1.03 (0.80–1.35)	1.08 (0.85–1.36)
Vertiginous syndrome	**1.39 (1.00–1.93)**	**1.67 (1.14–2.44)**	1.32 (0.89–1.95)
Depression	1.37 (0.98–1.92)	**1.46 (1.06–2.02)**	1.33 (1.00–1.76) ^‡^
Hypertension	1.08 (0.86–1.37)	1.10 (0.87–1.39)	1.08 (0.84–1.38)
Broad-spectrum antibiotics ^÷^	1.05 (0.85–1.30)	0.97 (0.76–1.24)	1.02 (0.78–1.33)
All anti-infectives ^L^	1.00 (0.83–1.20)	0.94 (0.78–1.14)	0.98 (0.75–1.28)

^a^ Adjusted for age, gender, and part of the year during which a patient was registered at a general practice. ^b^ Age ≥40 years, defined as any patient with asthma or COPD. Abbreviations: OR, odds ratio and CI, confidence interval. * *p* < 0.01. ^‡^ *p* = 0.01. ^÷^ ATC codes “J01CA04” (amoxicillin) + “J01AA02” (doxycycline). ^L^ ATC group “J” (anti-infectives for systemic use).
